# How climate impacts the composition of wolf‐killed elk in northern Yellowstone National Park

**DOI:** 10.1111/1365-2656.13200

**Published:** 2020-03-27

**Authors:** Christopher C. Wilmers, Matthew C. Metz, Daniel R. Stahler, Michel T. Kohl, Chris Geremia, Douglas W. Smith

**Affiliations:** ^1^ Center for Integrated Spatial Research Environmental Studies Department University of California Santa Cruz CA USA; ^2^ Yellowstone Center for Resources Yellowstone National Park WY USA; ^3^ Wildlife Biology Program Department of Ecosystem and Conservation Sciences University of Montana Missoula MT USA; ^4^ Department of Wildland Resources and Ecology Center Utah State University Logan UT USA

**Keywords:** age structure, *Canis lupus*, climate change, predator–prey dynamics, prey selection

## Abstract

1. While the functional response of predators is commonly measured, recent work has revealed that the age and sex composition of prey killed is often a better predictor of prey population dynamics because the reproductive value of adult females is usually higher than that of males or juveniles.
2. Climate is often an important mediating factor in determining the composition of predator kills, but we currently lack a mechanistic understanding of how the multiple facets of climate interact with prey abundance and demography to influence the composition of predator kills.
3. Over 20 winters, we monitored 17 wolf packs in Yellowstone National Park and recorded the sex, age and nutritional condition of kills of their dominant prey—elk—in both early and late winter periods when elk are in relatively good and relatively poor condition, respectively.
4. Nutritional condition (as indicated by per cent marrow fat) of wolf‐killed elk varied markedly with summer plant productivity, snow water equivalent (SWE) and winter period. Moreover, marrow was poorer for wolf‐killed bulls and especially for calves than it was for cows.
5. Wolf prey composition was influenced by a complex set of climatic and endogenous variables. In early winter, poor plant growth in either year *t* or *t *− 1, or relatively low elk abundance, increased the odds of wolves killing bulls relative to cows. Calves were most likely to get killed when elk abundance was high and when the forage productivity they experienced in utero was poor. In late winter, low SWE and a relatively large elk population increased the odds of wolves killing calves relative to cows, whereas low SWE and poor vegetation productivity 1 year prior together increased the likelihood of wolves killing a bull instead of a cow.
6. Since climate has a strong influence on whether wolves prey on cows (who, depending on their age, are the key reproductive components of the population) or lower reproductive value of calves and bulls, our results suggest that climate can drive wolf predation to be more or less additive from year to year.

## Graphical abstract

The impacts of large carnivores on their prey are importantly impacted by the composition of the prey killed. Here the authors show that climate and elk population size interact in important ways to determine when wolves kill cows, bulls or calves.

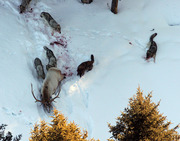

## 1 INTRODUCTION

The effects of large predators on the abundance of their ungulate prey populations are much debated in ecology. While Lotka–Volterra type predator–prey models often predict some top‐down regulation of prey populations by predators, empirical results vary widely. Some studies show strong regulatory effects of predators on prey, whereas others reveal little to no impact (Melis et al., 2009; Sinclair, Mduma, & Brashares, 2003), even within the same population (Wilmers, Post, Peterson, & Vucetich, 2006). Much research on large predators has focused on measuring their functional response (i.e. how kill rate varies with prey abundance) because this is the key predator‐driven element of simple Lotka–Volterra predator–prey models. There appears to be little correspondence, however, between a predator's kill rate and the population dynamics of their prey (Vucetich, Hebblewhite, Smith, & Peterson, 2011). This likely results, at least partially, from the exclusion of sex and age structure—in fact, recent work indicates that predator kill composition can have a much larger impact on understanding a predator species' effect on prey population dynamics than kill rates (Gervasi et al., 2012). When predators tend to kill low reproductive value prey such as juveniles or males, their impacts on prey population growth are small compared to when they more commonly kill prime‐age adult females with high reproductive value (Gervasi et al., 2012; Hoy et al., 2015). In order to more fully understand how predators impact their prey populations, a more detailed understanding of why predators kill different sex and age classes of their prey is needed.

Climate often mediates predator–prey interactions from both the bottom‐up by impacting the nutritional condition of prey and from the top‐down by mediating predator behaviour. In North America, for instance, wolf *Canis lupus* kill rates on elk *Cervus canadensis* typically increase from early to late winter as prey nutritional condition declines (Metz, Smith, Vucetich, Stahler, & Peterson, 2012). Additionally, deep snow leads to larger wolf packs with consequently higher kill rates on moose *Alces alces* (Post, Peterson, Stenseth, & McLaren, 1999), and tall grass that grows following heavy rains in Africa provides better predator stalking cover and allows lions *Panthera leo* to increase their kill rates on wildebeest *Connochaetes taurinus* (Packer et al., 2005). The composition of predator kills (i.e. prey composition) is also impacted by climate but is less well understood. Prey size is well known to influence vulnerability to predation (Sinclair et al., 2003), and this vulnerability can be altered by climatic factors. For instance, the composition of wolf‐killed elk has been shown to vary along a gradient of snow depth, with calves being selected for at intermediate snow depths (Huggard, 1993), but it was unclear in this study why calves were not also selected for at lower snow depths. As climate changes, a better understanding of the various mechanisms by which it mediates prey composition will be important to predicting ecological dynamics, as well as for managing and conserving ungulate and predator populations.

Summer plant productivity and winter snow conditions are the key climatic variables that are likely to influence prey nutritional condition and predator hunting success, and thus prey composition, in northern climes. Ungulates in mountainous and northern latitudes typically put on weight during the plant growing season (Parker, Barboza, & Gillingham, 2009) that occurs from late spring to early fall. At the end of the plant growing season, the nutritional condition of the prey population may vary from year to year depending on the forage conditions that occurred that year (Middleton et al., 2013) and possibly the previous year (e.g. the importance of lagged effects). Nonetheless, juveniles and females enter winter in their best annual nutritional condition, but many males do not because breeding‐age males compete for females during the fall rut (Mysterud, Langvatn, & Stenseth, 2004; Parker et al., 2009). These males expend significant energy competing for females while also decreasing their foraging efforts, resulting in at least some males (i.e. the breeding males) entering winter in suboptimal nutritional condition. All sex and age classes of ungulates then decline in their condition throughout winter as low‐quality food intake cannot keep pace with energetic demands (Parker et al., 2009). Deep or dense snows can accelerate this process by limiting access to forage and increasing the energetic costs of movement (Parker et al., 2009).

Yellowstone National Park (YNP) provides a natural laboratory in which to test the impacts of climate on prey composition because of its well‐studied large carnivore and herbivore populations. Climate change in YNP has already resulted in shorter winters with consequent impacts on temporal patterns of elk mortality (Wilmers, Crabtree, Smith, Murphy, & Getz, 2003; Wilmers & Getz, 2005), as well as increased drought that can diminish the efficacy of green wave surfing and reduce pregnancy rates in migratory elk (Middleton et al., 2013; Wilmers & Levi, 2013). In order to develop a more complete picture of how variability in climate drives this predator–prey system, we documented the age and sex of wolf‐killed elk in early and late winter when elk are near their best and worst nutritional condition, respectively, across 20 winters in northern YNP.

We predicted that elk demographics would be an important determinant of vulnerability to predation by wolves under differing climatic regimes. Calves are small in body size—making them generally more vulnerable to predation by wolves. As well, because of the high demands of growth, calves are less able to accumulate high fat reserves during summer. Cows are much larger than calves, which makes them generally less vulnerable to predation than calves and allows them to accumulate substantial fat reserves going into winter. Although bulls are much larger still than cows, participating in the aforementioned fall rut substantially decreases the nutritional condition of some bulls. Based on these interactions between elk life history and climatic conditions, we hypothesized that the composition of wolf‐killed elk would be largely driven by variation in summer productivity and winter snow accumulation. For instance, bulls might be more vulnerable to predation by wolves, and therefore more likely to be killed in early winter following summers of below average forage productivity. Here then we analysed how summer and winter climate conditions interacted with elk demography to influence wolf prey composition. Because of the long‐term nature of our dataset and the detailed counts of wolves and elk over the same period, we also included in our analysis whether wolf prey composition was affected by the lagged effects of climate, elk abundance and wolf pack size. We were unable to evaluate prey selection through the inclusion of calf, cow and bull relative abundance in our models because these data were not available for all years. But cows were always the most abundant elk demographic class (on average, ~69% of the northern Yellowstone elk population during years of our study for which data were available; Northern Yellowstone Cooperative Wildlife Working Group, unpubl. data), with their proportional availability also tending to increase as elk population abundance declined.

## 2 MATERIALS AND METHODS

### 2.1 Data collection

We collected data on the type of prey killed by wolves as part of a long‐term research programme on wolf ecology that began with the reintroduction of wolves to YNP in 1995 (Bangs & Fritts, 1996). The dataset for this analysis runs through March 2017, although we excluded data from the winters of 2013–2014 and 2014–2015 because of missing productivity data (see Section 2.2 below). Each year during the study, we used aerial‐ and ground‐based observations to intensively monitor two or three wolf packs for 1 month in both early winter (approximately 15 November–14 December) and late winter (approximately 1–30 March) on the Northern Range of YNP (*n* = 17 total packs over 40 unique study periods; see Martin et al., 2018 for further details). Except for one pack during one 30‐day period (Junction Butte during early winter 2012), we maintained at least one VHF collar on a wolf in each pack, thus allowing us to find each pack on a nearly daily basis using radio telemetry. Pack size was determined through our aerial‐ and ground‐based observations. If the wolves were found feeding on a kill, we recorded the species and age–sex class (calf, yearling, adult female and adult male). Although wolves have increasingly fed on bison in northern YNP (Metz, Smith, Stahler, Vucetich, & Peterson, 2016), wolves' use of bison has primarily occurred through scavenging. Direct killings by wolves have still focused on elk (Tallian et al., 2017) and we therefore focused our analyses only on elk. Through observations and/or necropsying of each carcass, we determined whether the elk was killed or scavenged by wolves (see also Metz et al., 2012), and only included those elk killed by wolves. Additionally, we collected a sample of elk femur marrow when available, and measured the percentage of fat therein using the dry‐weight method (Peterson, Allen, & Dietz, 1982).

### 2.2 Analysis

We divided wolf‐killed elk into three demographic categories; calves (0–1 years old), cows (females 2+ years old and all yearlings) and bulls (males 2+ years old). We included yearlings (*n* = 33) in the cow group because yearlings reside in cow‐dominated groups and their frequency among wolf‐killed elk was quite similar to that of prime‐aged cow elk as reported by Wright, Peterson, Smith, and Lemke (2006; Figure 3). We also tested whether our choice to include yearlings in the cow demographic group would affect our analysis by dropping yearlings from the analysis entirely and rerunning the analysis. The exclusion of yearlings made almost no appreciable difference in the results so we decided to keep them in to augment our sample size and reduce standard errors ever so slightly. In total, we detected and included 1,050 wolf‐killed elk (note that we removed 47 elk from the winters of 2013–2014 and 2014–2015 explained below). In order to understand whether wolf prey composition tracked prey nutritional condition, we first analysed the condition of each elk's bone marrow that we had samples for (*n* = 645). Bone marrow fat percentages below 85% are highly correlated with body fat (Cook et al., 2001), and animals below 70% are known to be in relatively poor nutritional condition (Ransom, 1965). As such, bone marrow fat gives us an estimate of how nutritional condition varies among animals (Metz et al., 2012) and with climatic conditions. We classified elk as being in poor condition if marrow fat was <70% (coded as 0) or relatively better condition otherwise (coded as 1). We then used logistic regression to evaluate which of the following covariates influenced marrow condition—elk demographic *class* (i.e. *cow*, *bull* or *calf*), winter study period (*WSP* = 0 for early winter and 1 for late winter), the estimated number of elk (*elk*) wintering within the Northern Range of YNP (Tallian et al., 2017), the productivity of vegetation during the preceding summer (*prod*_t_), the productivity of vegetation during the summer 1 year prior (*prod_t_*_−1_) and the average snow water equivalent (*SWE*) for that month (see Appendix S1 for details on the calculation of *prod* and *SWE*). In 2013, satellite imagery was not available to calculate vegetation productivity data due to extensive cloud cover. This resulted in excluding data from the winter of 2013–2014 and 2014–2015 from our analysis. All covariates were checked for collinearity using variance inflation factors (VIFs). None of the covariates had VIFs exceeding 1.07, indicating that collinearity was not a problem. We also tested for interactions between each of the climate variables and either the number or demographic class of elk. We reasoned that different age–sex classes of elk might respond differently to variation in food productivity and/or the harshness of winter conditions. As well, we expected that competition among elk, as indicated by elk population size, might further modulate these responses. The covariates in our full model predicting marrow fat were thus *class*, *WSP*, *elk*, *prod_t_*, *prod_t_*_−1_, *SWE*, *elk* × *prod_t_*, *elk* × *SWE*, *class* × *prod_t_* and *class* × *SWE*.

In order to understand the conditions under which wolves kill one demographic group of elk over another, we modelled data on prey composition using multinomial regression, with cows serving as the base case. Conceptually, this is similar to conducting two logistic regressions, the first of calves coded as 1 versus cows coded as 0, and the second of bulls coded as 1 versus cows coded as 0. We included the same predictor variables as for the marrow analysis with the addition of wolf pack size (*pack size*). However, we modelled the two time periods separately because we expected wolf prey composition to vary substantially between early and late winter. The covariates in our full models were thus *elk*, *prod_t_*, *prod_t_*_−1_, *SWE*, *pack size*, *elk* × *prod_t_* and *elk* × *SWE*. For each period, we again checked for collinearity among our covariates, and again found no evidence (all VIFs were <1.07). While in the marrow analysis we could explore interactions between WSP and elk demographic class through an interaction effect, this was not possible in the prey composition analysis because elk demographic class was the dependent variable.

We fit the logistic regression model predicting marrow fat as poor or relatively better using the glm function in r with a binomial link. We fit the multinomial model predicting the demographic class of a wolf‐killed elk using the multinom function in the NNET package in r. In both the analyses, we calculated the Akaike information criterion (AIC) values of the full model and all subsets of that model using the dredge function in the r package mumin (Barton, 2019). We then based our inferences on model‐averaged parameters from all models (Burnham & Anderson, 2002). To evaluate the performance of the marrow model, we calculated the area under the receiver operator curve (ROC). For the multinomial regression of wolf prey composition, we similarly calculated the area under the ROC for logistic regression versions of the model comparing calves to cows and bulls to cows, respectively, following the guidance in Hosmer and Lemeshow (2000). Parameters for calculating ROC values were derived from the model averaging procedure described above. ROC values of 0.5 suggest no discrimination, those between 0.7 and 0.8 are considered acceptable discrimination and those between 0.8 and 0.9 are considered outstanding discrimination. All continuous covariates were standardized by subtracting off their mean and dividing by the standard deviation (Gelman & Hill, 2007). This transformation allows the direct comparison of coefficient values for covariates that are measured on different scales. Once standardized in this way, a one‐unit change in the value of the coefficient, translates into a 1‐standard deviation change in the predictor.

## 3 RESULTS

**FIGURE 1:**
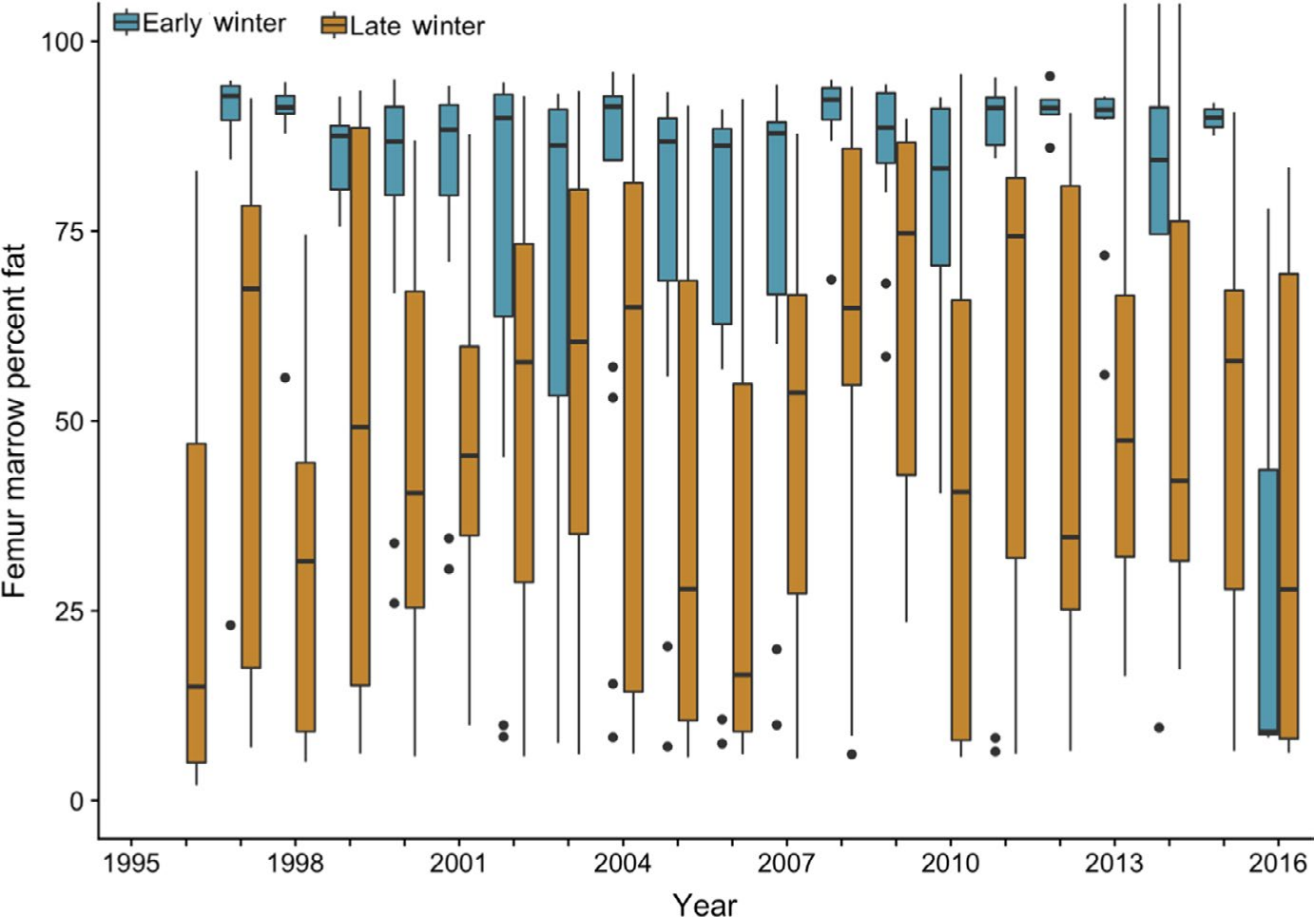
Box whisker plots of per cent fat in the femur marrow of individual wolf‐killed elk during early and late winter. Year on the *x*‐axis refers to the year on 31 December (e.g. 1995 represents the winter of 1995–1996). Data for 30 wolf‐killed elk from 2013–2014 and 2014–2015 are displayed here, although these data were excluded from the analysis (Figure 2)

**FIGURE 2:**
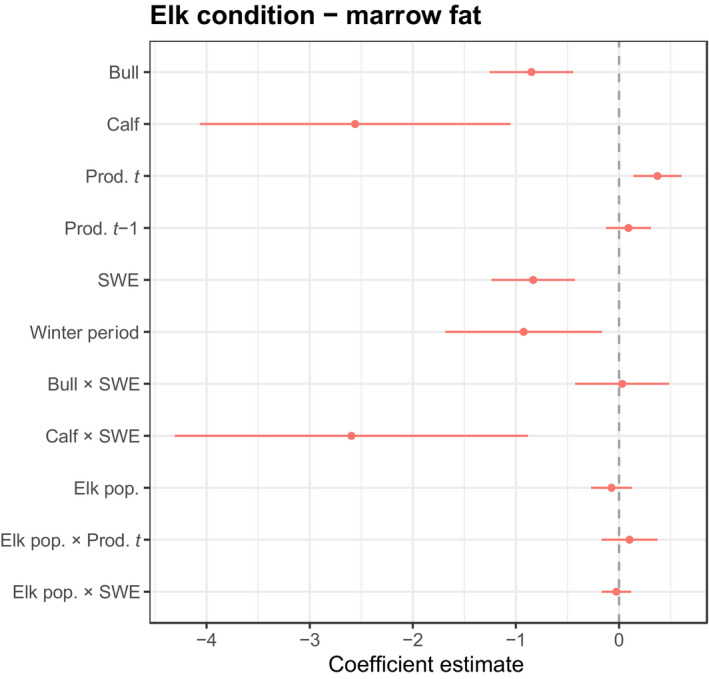
Determinants of elk nutritional condition. Model‐averaged standardized coefficient values ± 95% CIs from a logistic regression fitting elk demographic, wolf pack size and climatic variables to data on marrow fat (1 = healthy marrow and 0 = depleted marrow). Results for bulls and calves are relative to cows which served as the base case

We collected femur marrow from 645 wolf‐killed elk, 214 in early and 431 in late winter. The nutritional condition of these elk, as indicated by the percentage of marrow fat, declined substantially from early to late winter (Figure 1). The mean percent marrow fat (±*SE*) was 78.8 (±1.6) and 45.3 (±1.4) in early and late winter, respectively. Bulls, and especially calves, were more likely than cows to have depleted marrow fat when controlling for other covariates (Figure 2). Years with higher summer productivity led to higher marrow fat stores while increased snow water equivalent hastened the decline in elk nutritional condition. Snow water equivalent interacted strongly with the calf demographic class. As SWE increased, calves were more likely than cows to see a further decrease in marrow fat condition. Overall, our model using model‐averaged coefficients to predict marrow condition had excellent discrimination, with an area under the ROC of 0.84 (Hosmer & Lemeshow, 2000).

**FIGURE 3:**
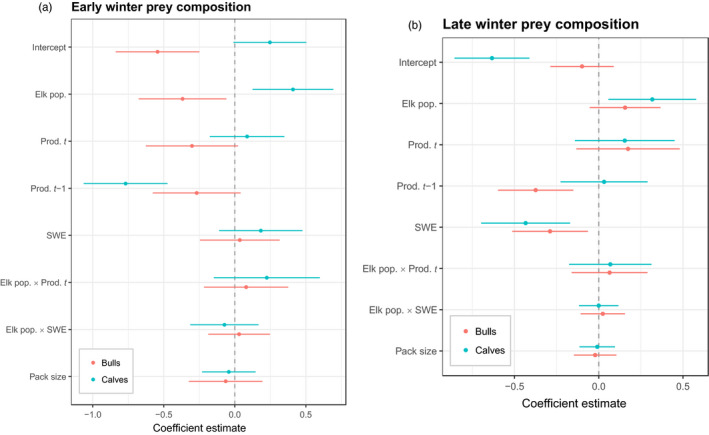
Wolf prey composition. Model‐averaged standardized coefficient values ± 95% CIs from a multinomial regression fitting elk population and climatic variables to data on elk demographic class (bull, cow and calf), with cow serving as the base case in (a) early winter and (b) late winter

**FIGURE 4:**
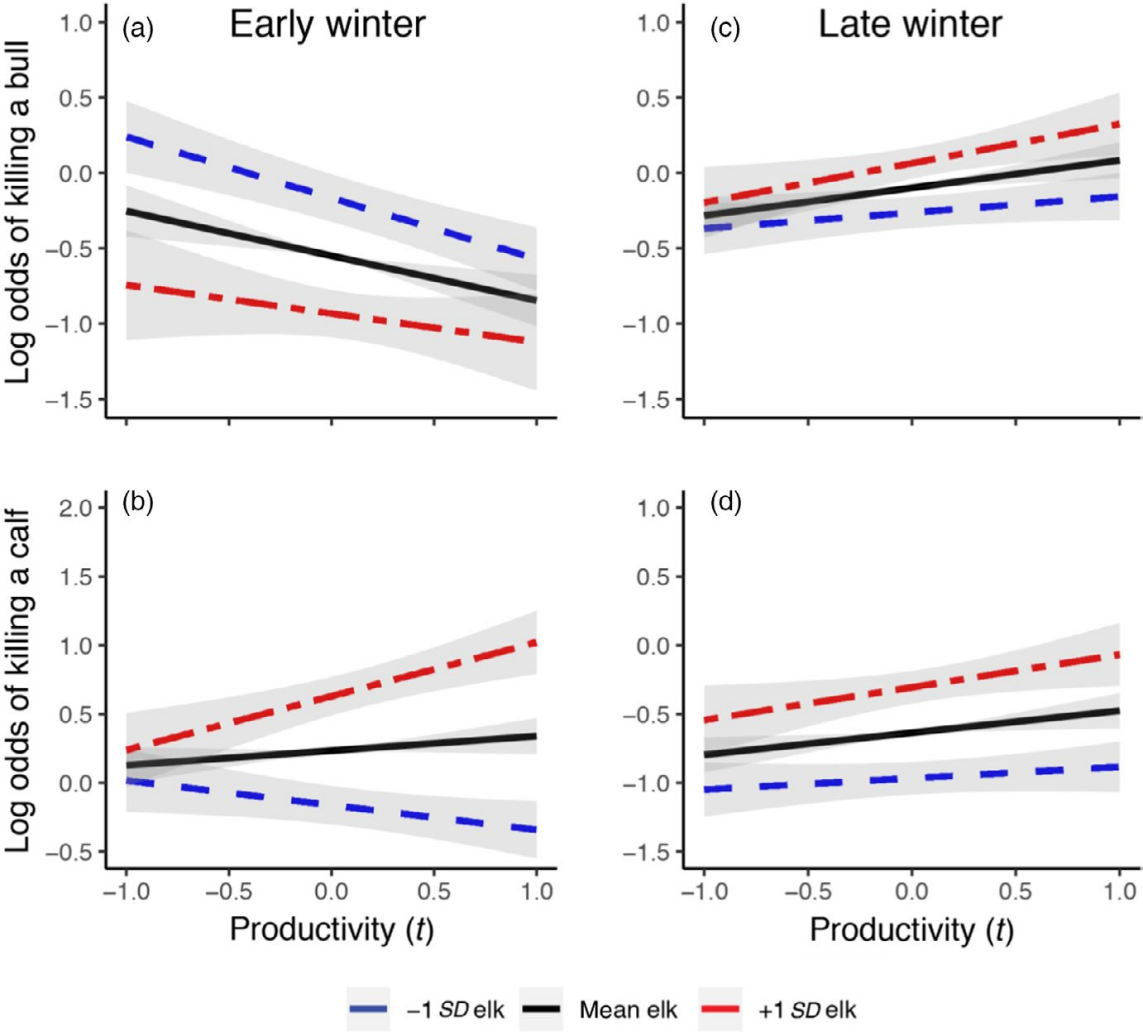
Plots of interaction effects from the multinomial regression fitting elk population and productivity in year *t* to data on elk demographic class, holding all other covariates to their mean values. We show predictions for the influence of productivity on the log odds of a kill at the mean (8,226 elk) and ±1 *SD* (±4,449 elk) of the elk population (mean and *SD* represent the mean and *SD* for our wolf‐killed elk data) for (a) bulls and (b) calves in early winter, and for (c) bulls and (d) calves in late winter. Shaded areas represent standard error intervals

We determined the demographic class of 1,050 wolf‐killed elk, 445 in early and 605 in late winter, respectively. In early winter, 150 (34%) were cows (i.e. cows and yearlings), 203 (46%) were calves and 92 (20%) were bulls, whereas in late winter 245 (40%) were cows, 136 (22%) were calves and 224 (37%) were bulls. Wolves were more likely to kill calves and less likely to kill bulls relative to cows in early winter (intercept coefficients in Figure 3a). The influence of summer productivity and elk population size had important modulating influences on this pattern. As elk population size increased, wolves became less likely to kill bulls relative to cows and more likely to kill calves (Figure 3a). As summer productivity in year *t* increased, wolves were less likely to kill bulls relative to cows in early winter (Figure 3a). Productivity also affected whether a calf was killed, but elk population size modulated the influence of productivity. At higher elk abundances, the odds of wolves killing a calf increased as productivity in year *t* increased (red line in Figure 4b), while at lower elk abundances, this relationship was reversed (blue line in Figure 4b). Summer productivity in year *t* − 1 also had a pronounced effect on the odds of wolves killing bulls and calves relative to cows (Figure 3a). The higher the productivity in year *t* − 1, the less likely wolves were to kill bulls and especially calves relative to cows. SWE in early winter did not appear to strongly influence prey composition. Wolf pack size was also not a factor in determining prey composition—which was perhaps not surprising given that wolf hunting success of elk does not improve beyond wolf group sizes of 4 (MacNulty, Smith, Mech, Vucetich, & Packer, 2012). Our logistic models using model‐averaged coefficients to predict that a wolf kill was a bull or calf, instead of a cow, in early winter each had areas under the ROC of 0.71 indicating acceptable discrimination (Hosmer & Lemeshow, 2000).

In late winter, wolves were generally less likely to kill calves and bulls relative to cows (intercept coefficients in Figure 3b). However, as elk population size increased the odds of wolves killing calves and bulls relative to cows also increased. As productivity in year *t* increased, so too did the odds that wolves kill bulls and calves in late winter (Figure 4c,d). Increased productivity in year *t* *−* 1, however, had little effect on wolves killing calves but significantly decreased the odds of wolves killing bulls (Figure 3b). More severe winters (as indicated by SWE) led wolves to kill fewer bulls and calves relative to cows. There was little evidence of important interactions between our climate variables and elk population size in late winter. Overall, our logistic models using model‐averaged coefficients to predict whether wolves killed a bull or calf in late winter had areas under the ROC of 0.63 and 0.65, respectively. This is a bit lower than the 0.7 threshold discussed by Hosmer and Lemeshow (2000) as being acceptable, but higher than the 0.5 mark which indicates no discrimination.

## 4 DISCUSSION

Our results are generally consistent with the idea that demographic characteristics and climate influence the vulnerability of elk to predation by wolves. Here we show that calves—most likely because they are smaller and can only accumulate limited fat reserves—and bulls—because they expend a portion of their fat reserves during the fall rut—are more susceptible to the effects of poorer forage conditions than cows. These observations are reflected in the fact that bulls and calves were more likely to have depleted marrow than cows (Figure 2). This vulnerability to climate then influenced the relative odds of an individual from a demographic group getting killed by wolves. The consequence of the interactions between climate and demographics is markedly different, however, between early winter when elk are in generally good condition and late winter when elk are in relatively poor condition (as reflected in our marrow data) and snow has also accumulated. By late winter, winter severity is the key climatic force.

In early winter, prey composition was driven largely by summer productivity and elk abundance. That snow was not an important factor is unsurprising because snow has only just started to accumulate. Elk abundance had a strong negative effect on the odds that wolves killed a bull relative to a cow so that the odds decreased as elk abundance increased. Interestingly, the odds of wolves killing bulls increased following summers with poor forage conditions (Figure 3a). Good plant productivity in year *t* − 1 also reduced the odds of wolves killing bulls in early winter (Figure 3a). This suggests that bulls' large body size and climate‐induced forage conditions interact to induce a time lag in their vulnerability to wolf predation—it takes 2 years of good forage for bulls to reach an optimal condition where they are best able to overcome the annual demands of the rut and then least vulnerable to predation by wolves during early winter.

Wolf predation on calves in early winter also varied importantly with both productivity and elk abundance but the relationships were more complex than those for bulls. In years with good plant productivity and many elk, wolves were much more likely to kill calves than cows (Figures 3a and 4b). If all elk demographic groups are in good shape when forage productivity is high, wolves are more likely to kill calves since their smaller body size makes them the most inherently vulnerable prey class. At low elk densities, however, we see the opposite pattern—namely that as productivity increases, wolves become less likely to kill calves relative to cows (Figure 4b). This could simply be a function of lower calf:cow ratios that often occur when elk abundance is lower. Unfortunately, we did not have sufficient data on the relative abundance of cows, bulls and calves so our inferences are limited in this case.

The largest impact of climate on calves getting predated in early winter was mediated by maternal effects. Previous research has shown that maternal condition influences calf metatarsal length, a proxy for body size in ungulates (Palsson & Verges, 1952). The better forage conditions were the summer immediately prior to the calf being in utero (i.e. at *t* − 1) in our study, the lower the odds that calves were killed in early winter by wolves (Figure 3a). This suggests that the forage conditions that mothers experience in the summer immediately preceding their pregnancy in winter influences whether their calves will be predated on by wolves during their first early winter (i.e. the maternal body condition carry over hypothesis; Lukacs et al. (2018).

In late winter, elk condition is highly variable among individuals and years (Figure 1). All animals lose weight over the course of the winter, with the body condition that an individual reaches at the end of winter being influenced both by their condition at the onset of winter (Cook et al., 2013) and winter severity (Loison & Langvatn, 1998). Calves in particular are highly vulnerable to harsh winters (as in other ungulate species; e.g. Cederlund, Sand, & Pehrson, 1991), and we observed a strong interaction between being a calf and SWE on marrow condition (Figure 2). Yet, our prey composition results for late winter reveal that as SWE increases or productivity declines, calves and bulls are less likely to be killed than cows (Figure 3b). The influence of SWE was especially clear and strong—this is likely because more snow makes all elk easier for wolves to kill (Huggard, 1993), and cows are the most abundant demographic class on the landscape. That cows are more likely to be killed with increased SWE, in combination with the knowledge that cows are also more likely to be killed as forage productivity declines (Figure 3b), suggests that a harsher climate levels the playing field—that is, with all animals highly vulnerable, predation on cows during late winter increases in comparison to predation on bulls and calves because cows are most abundant.

Taken together, our results provide insight into how climate and predation influence ungulate population dynamics. The relative body size of calves, cows and bulls sets a baseline of vulnerability to predation by wolves that is then modified by climate and elk relative abundance. As plant growing conditions worsen due to drought conditions or a shorter and more synchronized green wave (Wilmers et al., 2013), wolves increasingly prey on bulls in early winter (Figure 4b). Because bulls are of low reproductive value, this suggests that wolf predation in early winter, particularly at low elk densities and in a landscape of poor forage conditions (Figure 4b), is less additive than it would be if they instead selected for higher reproductive value of cows. Calves may also be of lower reproductive value than cows, depending on the gender of the calf and the age of the cow used for comparison. Unfortunately, we were not able to further partition calves by gender and cows by age. However, the average age of a wolf‐killed cow elk in our dataset was 14.7 years old. This is very close to the age at which cows have already started to senesce such that their reproductive value roughly matches that of a female calf (Wright et al., 2006). Assuming a 50–50 birth sex ratio for elk, no selection by wolves for calf gender and that male calves has a reproductive value near zero, the expected reproductive value of a wolf‐killed calf should then be about half that of an average wolf‐killed cow. As such, a preference by wolves for killing calves over cows in early winter may also decrease the degree to which predation is additive. In late winter, the picture is more complex. At high elk abundances (i.e. at +1 *SD* of the elk population or around 12,700 individuals) following good summers, wolves are more likely to prey on calves and bulls than at low elk abundance or when conditions are poor, thus allowing higher reproductive value of cows to live, reproduce and give birth to larger calves. Independent of density‐dependent effects (e.g. decreased pregnancy rates; Taper & Gogan, 2002), this would help a large population get larger still. At low elk population abundances (i.e. at −1 *SD* of the elk population or around 3,800 individuals) following poor summers and during harsh winters, however, cows are more likely to get predated, thus possibly preventing a small population from growing. As such, the combined effects of summer productivity, winter severity and elk population density interact in complex ways to potentially impact elk population dynamics.

Our work underscores the importance of interactions between climate, forage and predation that have been shown to impact elk population dynamics across the northwestern United States (Brodie et al., 2013; Griffin et al., 2011; Lukacs et al., 2018). Although these meta‐analyses have established these factors as being important drivers of elk survival and recruitment, there still exists a gap in our mechanistic understanding of how climatic factors affect the use of various prey types by predators, including wolves. Our work helps to fill that gap and indicates that these climatic drivers interact in complex and sometimes counterintuitive ways with elk life history, nutritional condition and population density to influence patterns of wolf‐induced elk mortality. These complex interactions suggest that the degree to which wolf predation is compensative or additive likely depends on not only prey abundance but also a suite of environmental and demographic factors.

## AUTHORS' CONTRIBUTIONS

D.R.S., C.C.W. and M.C.M. conceived the ideas and designed methodology; D.R.S., M.C.M., M.T.K., D.W.S. and C.G. collected the data; C.C.W., M.C.M., C.G. and M.T.K. analysed the data; C.C.W. led the writing of the manuscript. All authors contributed critically to the drafts and gave final approval for publication.

## Supporting information

Appendix S1

## Data Availability

Data available from the Dryad Digital Repository: https://doi.org/10.7291/D1W093 (Wilmers et al., 2020).
