# Supplementary material for: How climate impacts the composition of wolf‐killed elk in northern Yellowstone National Park
Source: J Anim Ecol. 2020 Mar 27;89(6):1511–9. doi: 10.1111/1365-2656.13200 (PMC7317765; doi:10.1111/1365-2656.13200)
Supplement: Supplementary file 1 — Appendix S1 [file JANE-89-1511-s001.docx]

**Appendix S1. Creating climatic covariates.**

Here we provide supplementary information related to the climatic covariates used in the marrow and prey composition analyses. We used elk distribution during summer and winter to define the areas over which we estimated plant productivity and Snow Water Equivalent (SWE) in summer and winter respectively.

*Elk distribution during summer and winter.*

We created 95% population-level home ranges for northern Yellowstone elk for ‘summer’ (April–October) and ‘winter’ (November–April) (Figure S1) when northern Yellowstone elk are generally ‘on’ and ‘off’ of their summer range, respectively, using kernel density estimation (KDE). The KDE was estimated using Plugin Estimation from the KernSmooth package (R 3.0.2).

Because we were interested in a population-level home range, we included locations for all Very High Frequency (VHF) and Global Positioning System (GPS) radio-collared adult female elk (*n* = 25) from northern Yellowstone from March 2000 – October 2014. To limit the influence of GPS locations, we reduced the GPS data to allow a maximum of one location per day per individual. In total, we used 34,548 locations (VHF: 6,425; GPS: 28,123), of which 12,643 were from summer, and 21,905 from winter. The number of locations (i.e., individual days) for an individual elk ranged from 1 to 983.


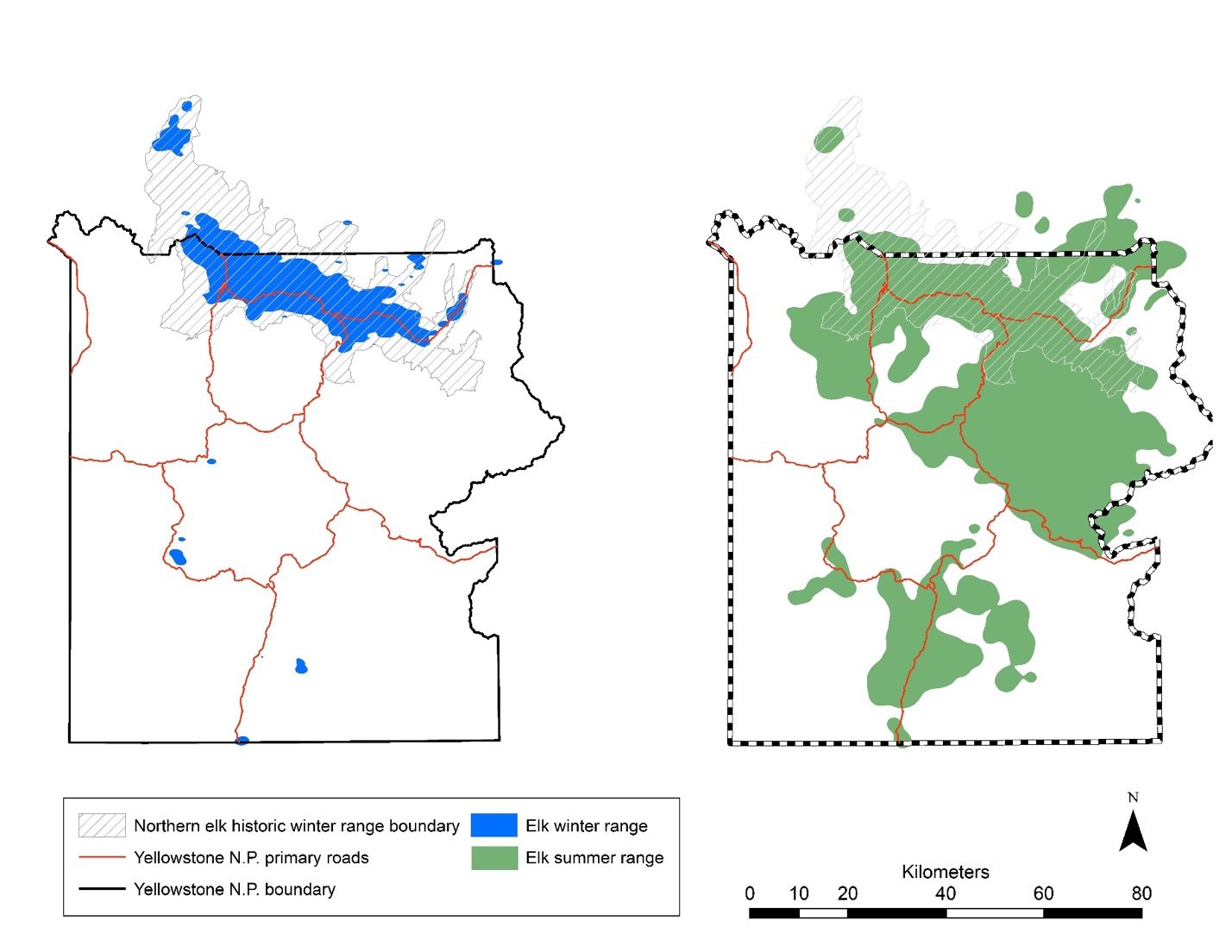


Figure S1 - Elk winter and summer ranges

*Forage production during summer*

Standing crop (grams m^-2^) estimates were generated for October 15 of the year using a simulation model that predicted 30-m^2^ resolution surfaces of modeled monthly net primary productivity from NASA’s Carnegie-Ames-Standford-Approach (CASA; Potter et al. 2007, Geremia et al. 2014). Estimates were computed as the sum of all monthly CASA net primary production (NPP) values multiplied by 2 for a carbon to dry biomass conversion factor. Total dry biomass is converted into aboveground forage biomass by a conversion factor of 0.45 for non-riparian or riparian grass, and 0.3 for riparian shrub. Monthly NPP were estimated using solar insulation, temperature, precipitation, and the Normalized Differential Vegetation Index (NDVI). A single NDVI image was used each year at the time of peak greenness of the growing season (Potter and Klooster 1999, Crabtree et al. 2009, Huang et al. 2010). NPP values were only available in non-forested areas and not available when annual NDVI images contained clouded regions. Areas with clouds or standing water (e.g., lakes, marshes, rivers) were eliminated by processing NDVI imagery to remove values less than 0.00 – 0.20 and greater than 0.90 – 1.00 depending on the quality of the growing season. Standing crop values were averaged across the ‘summer’ elk KDE. Standing crop values did not account for summer offtake (by herbivores), but nonetheless served as a reasonable surrogate for the quality of the growing season. In 2009, clouds over the southern and western parts of the study area likely lowered standing crop estimate. In 2012, resolution of NDVI image was 250m^2^ whereas other years are based on an NDVI image with 30m^2^ resolution.

*Winter severity*

We used spatially-explicit predictions for Snow Water Equivalent (SWE) from a ‘snow model’ developed for Yellowstone National Park (Wockner et al. 2006) to characterize winter severity across the winter distribution of elk within YNP. Here, we first defined the spatial extent of our study area through the 95% ‘winter’ elk KDE, but defined the northern boundary as the northern boundary of YNP because most (i.e., 996 of 1,009) of the wolf-killed elk in our dataset were located within YNP. We then used this polygon to clip the weekly spatial predictions for SWE for each week that was included within the 30-day study period of interest. Finally, we took the mean pixel value across our study area for each week, and then the mean of those weekly values.

**Literature Cited**

Crabtree R, C Potter, R Mullen, J Sheldon, S Huang, J Harmsen, et al. 2009. A modeling and spatio-temporal analysis framework for monitoring environmental change using NPP as an ecosystem indicator. Remote Sensing of the Environment 113:1486–1496.

Geremia C, PJ White, RA Garrott, RL Wallen KE Aune, JT Treanor, et al. 2009. Demography of central Yellowstone bison: effects of climate, density and disease. in: Garrott RA, White PJ, Watson FGR, eds. The Ecology of Large Mammals in Central Yellowstone: Sixteen Years of Integrated Field Studies. San Diego: Elsevier. pp 255–279.

Geremia C, PJ White, JA Hoeting, RL Wallen, FGR Watson, D Blanton, et al. 2014. Integrating population- and individual- level information in a movement model of Yellowstone bison. Ecological Applications 24: 346-362.

Huang S, CS Potter, RL Crabtree, S Hager, P Gross. 2010. Fusing optical and radar data to estimate grass and sagebrush percent cover in non-forested areas of Yellowstone. Remote Sensing of the Environment 2:251–264.

Potter CS, SA Klooster. 1999. Dynamic global vegetation modeling (DGVM) for prediction of plant functional types and biogenic trace gas fluxes. Global Ecology and Biogeography Letters 8: 473–488.

Potter C, S Klooster, A Huete, V Genovese. 2007. Terrestrial carbon sinks for the United States predicted from MODIS satellite data and ecosystem modeling. Earth Interactions 11:1–21.

Watson FGR, WB Newman, JC Coughlan, RA Garrott. 2006. Testing a distributed snowpack simulation model against spatial observations. Journal of Hydrology 328: 728-734.

Wockner, G., Singer, F., Coughenour, M., & Farnes, P. Yellowstone Snow Model. Colorado State Univ., Fort Collins, CO (2006).
